# Hindbrain Ghrelin Receptor Signaling Is Sufficient to Maintain Fasting Glucose

**DOI:** 10.1371/journal.pone.0044089

**Published:** 2012-08-31

**Authors:** Michael M. Scott, Mario Perello, Jen-Chieh Chuang, Ichiro Sakata, Laurent Gautron, Charlotte E. Lee, Danielle Lauzon, Joel K. Elmquist, Jeffrey M. Zigman

**Affiliations:** 1 Division of Hypothalamic Research, Department of Internal Medicine, The University of Texas Southwestern Medical Center at Dallas, Dallas, Texas, United States of America; 2 Department of Pharmacology, University of Virginia, Charlottesville, Virginia, United States of America; 3 Division of Endocrinology & Metabolism, Department of Internal Medicine, The University of Texas Southwestern Medical Center at Dallas, Dallas, Texas, United States of America; 4 Department of Psychiatry, The University of Texas Southwestern Medical Center at Dallas, Dallas, Texas, United States of America; 5 Department of Pharmacology, The University of Texas Southwestern Medical Center at Dallas, Dallas, Texas, United States of America; University of Cordoba, Spain

## Abstract

The neuronal coordination of metabolic homeostasis requires the integration of hormonal signals with multiple interrelated central neuronal circuits to produce appropriate levels of food intake, energy expenditure and fuel availability. Ghrelin, a peripherally produced peptide hormone, circulates at high concentrations during nutrient scarcity. Ghrelin promotes food intake, an action lost in ghrelin receptor null mice and also helps maintain fasting blood glucose levels, ensuring an adequate supply of nutrients to the central nervous system. To better understand mechanisms of ghrelin action, we have examined the roles of ghrelin receptor (GHSR) expression in the mouse hindbrain. Notably, selective hindbrain ghrelin receptor expression was not sufficient to restore ghrelin-stimulated food intake. In contrast, the lowered fasting blood glucose levels observed in ghrelin receptor-deficient mice were returned to wild-type levels by selective re-expression of the ghrelin receptor in the hindbrain. Our results demonstrate the distributed nature of the neurons mediating ghrelin action.

## Introduction

Ghrelin is a peptide hormone secreted predominantly by a sparsely-distributed group of endocrine cells in the gastrointestinal mucosa [1]. Ghrelin was originally described as a potent growth hormone releasing signal, acting through the G-protein coupled receptor GHSR in the brain [1]. Studies since have revealed a wider role for ghrelin in numerous metabolism-related processes. Ghrelin is hypothesized to act as a meal initiator signal and fuel storage gauge, secreted at times of low nutrient availability [2], [3]. Not only does ghrelin potently stimulate food intake [4], [5], [6], but also the manifestation of various food-reward behaviors depends on ghrelin action in the central nervous system (CNS) [7], [8]. Ghrelin also raises blood glucose, which is particularly relevant during severe caloric restriction when it prevents marked hypoglycemia and death [9], [10], [11], [12], though this action of ghrelin is likely dependent on other yet to be identified genetic and environmental modifiers [13].

The sites of ghrelin action in the coordination of food intake and glucose homeostasis have yet to be definitively identified, but undoubtedly include direct action on one or more of several CNS sites implicated in the control of nutrient intake and blood glucose. For example, GHSRs are expressed within many nuclei comprising the medial basal hypothalamus, hindbrain, and midbrain, including sites already known to mediate food intake and glucose homeostasis [14]. Also, ghrelin can induce c-fos expression in many of these sites, suggesting its ability to activate their resident neurons, the end result of which could be changes to behaviors and processes controlling food intake and blood glucose [6], [15]. For instance, direct injection of ghrelin into the hypothalamic arcuate nucleus stimulates food intake while interference with signaling by arcuate AgRP/NPY neurons has the opposite effect [6], [16]. Ghrelin injection into the dorsal vagal complex of the hindbrain also induces feeding and recapitulates the induction of c-fos produced by intracerebroventricular ghrelin injection [17]. Increased food intake also occurs upon ghrelin injection into the ventral tegmental area (VTA) of the midbrain [18], [19], an effect lost in the ghrelin receptor null animals. The relative contribution of each of these central nuclei expressing GHSRs in the control of ghrelin-induced feeding, however, is debatable.

In this report, we describe the use of a Phox2b-Cre recombinase-expressing mouse line that permits the selective expression of GHSR in specific hindbrain nuclei implicated in the control of food intake and glucose homeostasis [5]. Outside of the hindbrain, the mouse remains null for the receptor, allowing us to test the sufficiency of ghrelin signaling in this area to modulate both feeding and glucose levels. Using this approach, we demonstrate that hindbrain ghrelin signaling is not sufficient to mediate the effects of ghrelin on acute food intake but is sufficient to normalize blood glucose following a fast.

## Materials and Methods

### Animal Husbandry

All procedures were conducted in accordance with the UTSW Institutional Animal Care and Use Committee guidelines and those of AAALAC. Furthermore, the UT Southwestern IACUC committee specifically approved the work reported in this current study. Mice were housed in a pathogen-free facility on a 12h light/dark cycle with ad libitum access to food and water unless specified otherwise. Male mice were used for all experiments. GHSR-null mice, which contain a loxP-flanked transcriptional blocking cassette within the endogenous GHSR alleles [5], and Phox2b-Cre mice [20] were both on a pure C57Bl/6J genetic background. Phox2b-Cre mice were mated to heterozygous GHSR-null animals, and then offspring hemizigous for Phox2b-Cre and heterozygous for the GHSR-null allele were crossed with mice heterozygous for the GHSR-null allele to produce study animals (wild-type, GHSR-null, GHSR-null/Phox2b). Mice hemizygous for Phox2b-Cre and wild-type for the GHSR allele showed no obvious phenotypic differences from wild-type animals [our unpublished observations and Scott et al. [20]], and thus, wild-type animals were used as controls in all reported experiments.

### In Situ Hybridization Histochemistry (ISHH)

The extent of GHSR expression resulting from Phox2b-Cre-dependent removal of the transcriptional blocking cassette as compared to that observed in wild-type mice was determined using single-label free-floating ISHH (n = 4), as described previously [7], [21].

### Ghrelin-Stimulated Food Intake

Six- to eight-week-old male mice were individually housed for 7 days prior to experimentation. Three days prior to experimentation, food was removed and replaced with 2 pellets of mouse chow placed in a petri dish on the cage floor. Mice were acclimated to the handling protocol for 3 days prior to s.c. injection of either saline vehicle or 2 µg/g mouse acyl-ghrelin (Pi Proteomics, Huntsville, Al). At 11 am on the test day, one chow pellet was weighed and placed in a petri dish on the cage floor followed by injection of saline into one half of the cohort and ghrelin into the other. Chow intake was followed for 3 hr. After experimentation, mice were singly housed for 7 days and the experiment repeated. Mice that received ghrelin during the first experiment received saline during the second trial and vice versa.

### Glucose Measurements

Fed and fasted (18 hr) glucose levels were monitored between 12–3 pm using a OneTouch Ultra glucometer and testing strips (LifeScan, Inc. Milpitas, CA). Blood was obtained from tail veins nicked using a disposable razor blade. For the fasting measurements, food was removed just prior to lights off (6 pm) the day preceding measurement of glucose.

### c-fos Expression Analysis

Mice were injected with acyl-ghrelin, as described above. Mice were subsequently transcardially perfused with formalin, and sectioned brains were stained using anti-c-fos antisera, as detailed previously [21].

### Data and Statistical Analyses

Brain sections were analyzed with a Zeiss Axioplan light microscope. Data are presented as average +/− SEM. Adobe Photoshop 7.0 was used to adjust only sharpness, brightness, and contrast as well as to combine selected images into plates. Comparisons of food intake following ghrelin or saline injection and of fasting glucose levels were performed using a 1-way ANOVA and Tukey’s post-hoc test.

## Results

### Phox2b-Cre-mediated Reactivation of GHSR Expression

**Figure 1:**
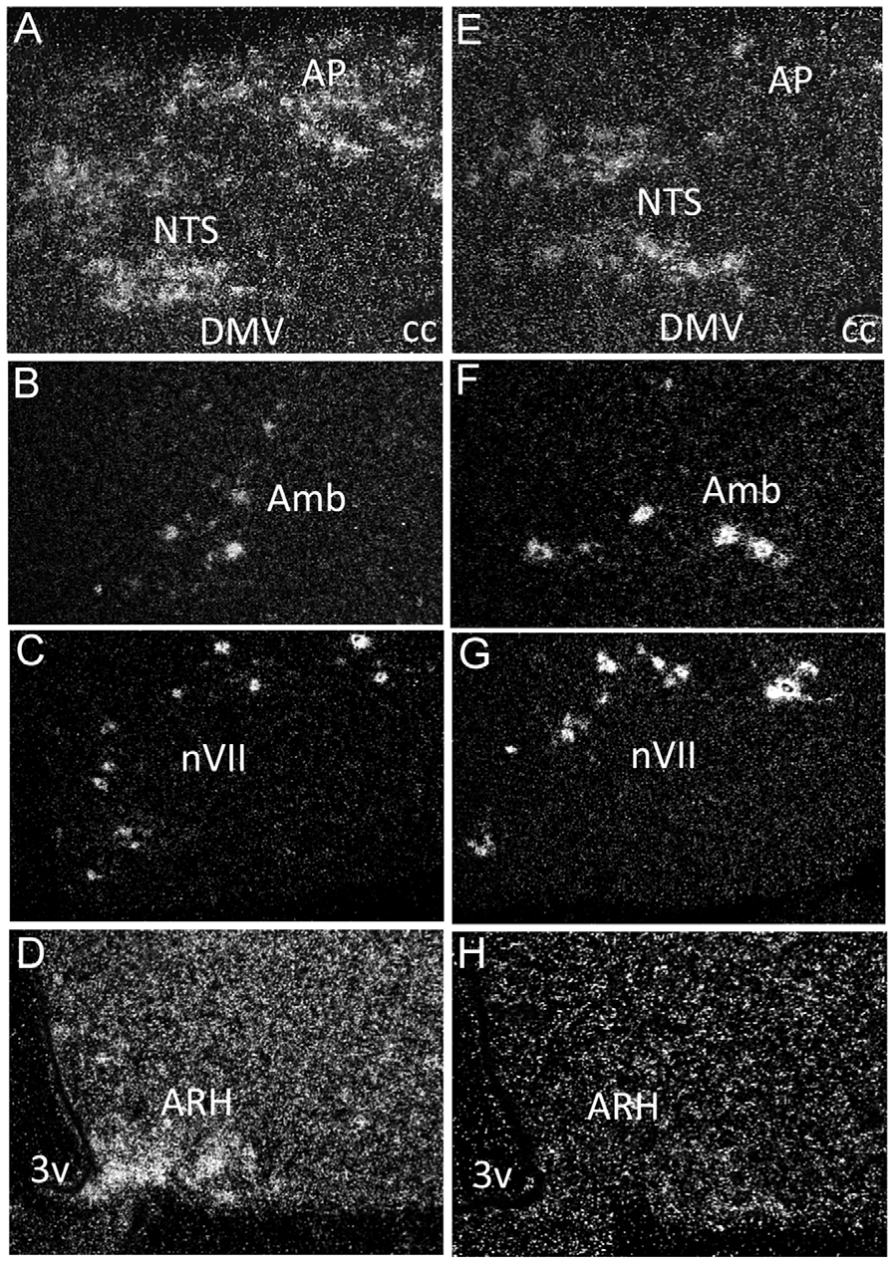
GHSR mRNA expression is restored selectively in the hindbrain in GHSR-null/Phox2b mice. Coronal sections of a representative wild-type mouse brain demonstrating expression in the nucleus of the solitary tract, area postrema and vagus motor nerve (A, NTS, AP, DMV), nucleus ambiguus (B, Amb), 7th nerve (C, nVII) and the arcuate nucleus of the hypothalamus (D, ARH). Coronal sections of a representative GHSR-null/Phox2b mouse brain demonstrating re-activated GHSR expression in all hindbrain nuclei (E-G) but not in the ARH (H).

To test the sufficiency of hindbrain ghrelin receptor expression in the mediation of ghrelin-stimulated feeding, we used a mouse model system that is conditionally null for GHSR (GHSR-null). These mice contain a loxP-flanked transcriptional blocking cassette inserted into intronic DNA upstream of the GHSR translational start codon, thus blocking GHSR expression [5], [7]. To re-express GHSR selectively in the hindbrain, we crossed the GHSR-null mice to a line of mice expressing Cre recombinase from the Phox2b locus of a bacterial artificial chromosome. These Phox2b-Cre mice express Cre selectively in the hindbrain, within brachial and visceral motor neurons and cells of the nucleus of the solitary tract (NTS) and area postrema [20], [22]. In characterizing the GHSR-null/Phox2b mice (which contain two GHSR-null alleles and Phox2b-Cre), we determined that GHSR was expressed in all hindbrain nuclei that had previously shown GHSR expression [14], at levels that appeared to recapitulate that of wild-type mice (Figure 1). In particular, just as was observed in wild-type mice, within GHSR-null/Phox2b mice, GHSR mRNA expression was observed by ISHH in all three components of the dorsal vagal complex [including the NTS, dorsomotor nucleus of the vagus nerve (DMV), and area postrema (AP)], nucleus ambiguus (Amb) and facial motor nucleus (nVII). Expression was absent from all other areas of the brain (including the arcuate nucleus) of GHSR-null/Phox2b mice (figure 1 and data not shown), demonstrating the desired and expected restriction of GHSR expression to the hindbrain.

### Induction of c-fos by Ghrelin in Mice with Hindbrain-selective GHSR Expression

Multiple studies have suggested that hindbrain neurons exhibit c-fos expression in response to exogenous ghrelin administration – both centrally (into the 4^th^ ventricle or directly into the hindbrain) and peripherally (including intravenously or intraperitoneally) [23], [24], [25], [26], [27], [28]. It also is known that hindbrain delivery of ghrelin via the 4^th^ ventricle does not induce c-fos in the arcuate nucleus or paraventricular nucleus as it does when administered into the forebrain ventricles or directly into those nuclei, suggesting the presence of partially independent forebrain and hindbrain circuits that respond to ghrelin [23], [25], [29]. However, it has not been determined definitively whether the induction of c-fos in the hindbrain by naturally-produced ghrelin in the periphery is the result of direct ghrelin action at the hindbrain or the result of indirect ghrelin action outside the hindbrain.

**Figure 2:**
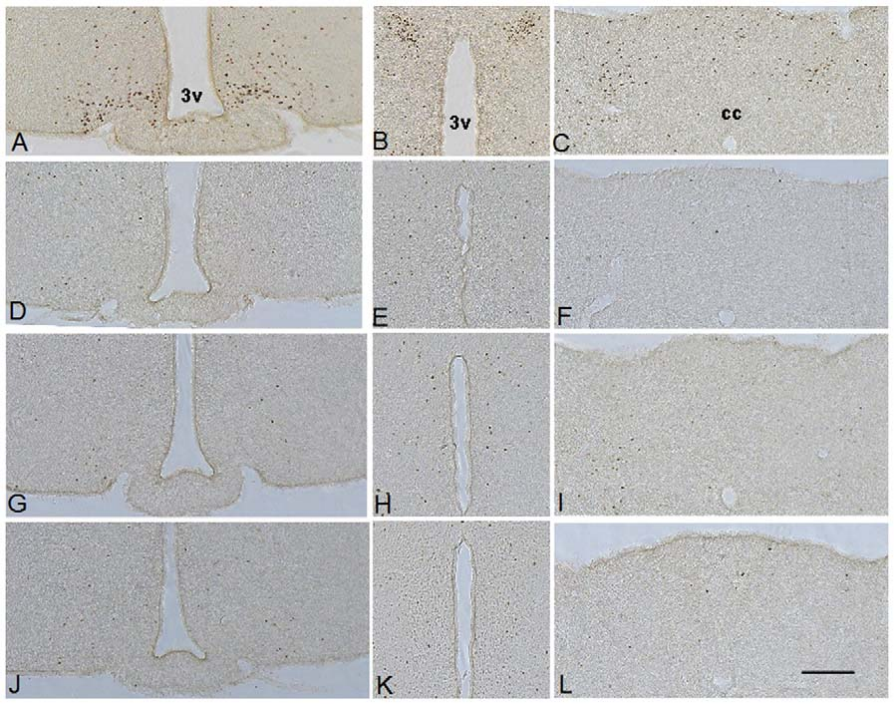
Phox2b-Cre-mediated GHSR re-expression does not restore CNS c-fos induction. Ghrelin (2 µg/g s.c.) induces robust c-fos expression in the arcuate nucleus (A) and the paraventricular nucleus (B) of the hypothalamus and in the area postrema and nucleus of the solitary tract of the hindbrain (C) in wild-type mice. Saline does not induce c-fos in the brains of wild-type mice (D-F). Ghrelin administration fails to induce c-fos expression in GHSR-null (G-I) and GHSR-null/Phox2b mice (J−L). (n = 3) 3v, third ventricle. cc, central canal.

As an initial test of the role of hindbrain GHSR receptor expression on the sensing of ghrelin produced in the periphery, we examined the induction of brain c-fos expression in response to administered ghrelin. A 2 µg/g BW subcutaneous dose of ghrelin was chosen because it can acutely induce food intake and certain antidepressant-like and food-reward behaviors and also can acutely elevate blood glucose [8], [12]. In wild-type mice, we observed significant c-fos expression in the hindbrain (AP and NTS) and hypothalamus (arcuate nucleus and paraventricular nucleus) (Figure 2). However, ghrelin administration failed to induce c-fos expression in GHSR-null/Phox2b mice above that of the basal levels observed in saline-injected wild-type mice or ghrelin-injected GHSR-null animals, indicating that hindbrain GHSR expression was not sufficient to mediate the shift in gene expression induced by the injection of ghrelin (Figure 2). The induction of hindbrain c-fos in wild-type mice, therefore, must be an indirect result of the actions of ghrelin outside of the hindbrain.

### Ghrelin-stimulated Food Intake is not Rescued by Hindbrain Expression of GHSR

**Figure 3:**
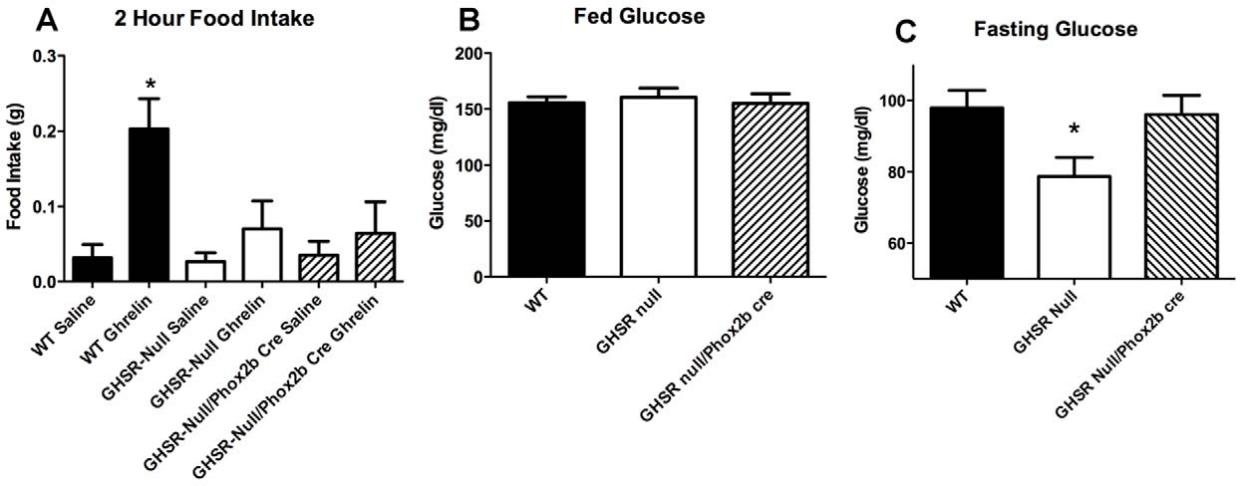
Phox2b-Cre-mediated GHSR re-expression fails to normalize ghrelin-stimulated feeding but restores fasting glucose levels. Ghrelin (2 µg/g s.c.) potently induces 2-hr food intake when compared to saline injection in wild-type but not in GHSR-null mice or in GHSR null/Phox2b cre mice. (n = 7, * = P<0.05 One-way ANOVA with Tukey’s post-hoc test) (A). Hindbrain-selective GHSR expression restores fasting blood glucose to that of wild type. (n = 25, * = P<0.05 1-way ANOVA with Tukey’s post-hoc test C).

We next addressed whether expression of GHSR in the hindbrain was sufficient to mediate an increase in food intake resulting from ghrelin administration. We tested this hypothesis by examining ghrelin-stimulated food intake in GHSR-null/Phox2b mice and their wild-type and GHSR-null control littermates. As mentioned, subcutaneous ghrelin at the 2 µg/g BW dose, as well as at lower doses, potently stimulates food intake over a two-hour period in wild-type mice [7], [8]. Thus, as expected, feeding was observed in wild-type mice during the first 2-hr period following subcutaneous ghrelin administration when compared to saline injection (Figure 3A). No effect of ghrelin was observed in the GHSR-null/Phox2b mice, as indicated by food intake after ghrelin injection that was identical to that of saline-injected wild-type animals and ghrelin–injected GHSR-null animals (Figure 3A). Thus, we conclude that hindbrain expression of GHSR is not sufficient to drive the feeding response to subcutaneously-administered ghrelin.

### Fasting Hypoglycemia is Rescued in Mice with GHSR Expression Limited to the Hindbrain

Several studies have demonstrated that ghrelin is required to maintain glucose homeostasis during fasting exists. For example, GHSR deletion lowers fasting blood glucose in mice exposed to an overnight fast; this greater fall in blood glucose has been associated with increased insulin sensitivity and reduced circulating glucagon [5], [7], [30], [31]. The fall in blood glucose observed upon interference with ghrelin signaling is exacerbated even further upon depletion of fat stores, as occurs with prolongation and intensification of the caloric restriction [9], [11], an action clearly modified by other genetic and environmental influences [13]. Thus, here we tested whether the hindbrain expression of GHSR was sufficient to rescue fasting glucose levels.

Although glucose levels were identical in *ad libitum*-fed wild-type, GHSR-null and GHSR-null/Phox2b mice (Figure 3B), following a fast, GHSR-null mice exhibited significant hypoglycemia compared to wild-type mice, as expected (Figure 3C). Notably, selective GHSR expression in the hindbrain was sufficient to normalize this relative hypoglycemia, as fasting glucose levels in the GHSR-null/Phox2b mice were significantly different from those in GHSR-null mice n = 25) p<0.05) and not significantly different from those in wild-type mice (Figure 3C).

## Discussion

In this study, we have employed a novel genetically-engineered mouse model with ghrelin receptor (GHSR) expression limited to the hindbrain to determine if such site-selective, hindbrain GHSR expression is sufficient to mediate ghrelin’s actions on food intake and blood glucose. With respect to intake of freely-available food, hindbrain GHSR expression was not sufficient to permit the characteristic orexigenic response to subcutaneous ghrelin administration observed in wild-type animals. With respect to the modulation of glucose homeostasis, hindbrain GHSR expression was sufficient to defend against the exacerbated fasting-induced fall in blood glucose that is otherwise observed in mice with global GHSR deficiency. Interestingly, although subcutaneous ghrelin administration induces c-fos in the hindbrain of wild-type animals, such does not occur when GHSR expression is limited to the hindbrain. Thus, hindbrain c-fos induction seems dispensable for ghrelin-dependent modulation of fasting glucose levels. These data help clarify the relevant sites of ghrelin receptor action in the brain in the modulation of food intake and blood glucose.

Several previous studies have implicated both the hypothalamus and the hindbrain as important CNS regions mediating ghrelin’s orexigenic actions. Among the many studies focusing on the hypothalamus, genetic ablation of both neuropeptide Y and agouti-related protein, which are normally co-expressed in a group of GHSR-expressing, arcuate neurons, was shown to completely abolish the acute orexigenic action of intraperitoneally-administered ghrelin [32]. Similarly, preventing release of the inhibitory neurotransmitter GABA from these arcuate NPY/AgRP neurons markedly attenuates acute food intake in response to intraperitoneal ghrelin [33]. In studies focusing on the hindbrain, direct microinjection of ghrelin into the dorsal vagal complex (which includes GHSR-containing neurons in the AP, NTS and dorsal motor nucleus of the vagus) was shown to stimulate food intake, at a dose lower than the lowest effective dose shown to induce food intake upon microinjection into the arcuate nucleus [16], [17]. Delivery of ghrelin to the dorsal vagal complex and the rest of the caudal brainstem, via injection into the fourth ventricle, also acutely increases food intake, number of meals, and speed of first meal onset; such is comparable to those changes elicited by ghrelin infusion into the third ventricle, in which ghrelin is exposed additionally to the arcuate nucleus [17]. Interestingly, total subdiaphragmatic vagotomy blocks the orexigenic actions of ghrelin upon its peripheral administration, and such is thought to occur independently of vagal afferent signaling [34], [35], [36].

While these latter studies indicate that GHSR-containing hindbrain neurons have the capacity to mediate ghrelin-stimulated food intake and that intact vagal signaling may be required for ghrelin’s overall acute effects on food intake, the data here demonstrates that direct sensing of ghrelin by GHSR-expressing hindbrain neurons is not sufficient on its own to mediate these acute orexigenic effects of ghrelin. Conversely, hindbrain ghrelin signaling does contribute to the maintenance of fasting glucose levels, as evidenced by the recapitulation of the usual (e.g. observed in wild-type mice) blood glucose response to an overnight fast by hindbrain-selective GHSR expression.

These data complement our prior studies investigating the effects of tyrosine hydroxylase-Cre-driven GHSR expression, in which GHSR expression occurs selectively in catecholaminergic (predominantly dopaminergic) neurons, such as those in the VTA (Chuang et al., 2011a). Notably, and unlike with Phox2b-Cre-driven hindbrain GHSR expression, catecholaminergic GHSR expression was sufficient to partially rescue ghrelin-stimulated acute food intake, while also fully restoring the ability of administered ghrelin and chronic stress to modulate food reward (Chuang et al., 2011a). Also unlike with the hindbrain-selective GHSR expression observed here, fasting blood glucose levels were not rescued by selective GHSR expression in catecholaminergic cells (Chuang et al., 2011a).

Regarding the involvement of ghrelin with the control of blood glucose, it most likely is the case that ghrelin’s full effect includes direct actions not only on the hindbrain, but also on other CNS sites and peripheral organs that affect glucose homeostasis. For instance, ghrelin has the capacity to directly bind to GHSRs on pancreatic alpha cells and beta cells, leading to stimulation of glucagon release and inhibition of insulin release, respectively, both of which would tend to raise blood glucose levels [37]. The ability of ghrelin to potently stimulate growth hormone (GH) secretion is potentially important in blood glucose homeostasis, as evidenced by the marked hypoglycemic and insufficient GH responses to severe caloric restriction in mice lacking ghrelin, and the correction of the marked hypoglycemia by pharmacologic preservation of the usual GH response [9], [11]. Interestingly, a recent report demonstrates that this role of ghrelin may be dependent on several modulating factors [13], complicating the investigation of ghrelin function. Thus, further studies will be required to determine in just what settings and in just what manner GHSR-expressing hindbrain neurons coordinate with other directly ghrelin-responsive neurons, pancreatic islet cells and pituitary cells to modulate blood glucose. Additional studies also will be needed to more extensively describe the integrated neuronal circuitry through which ghrelin contributes to food intake as well as to other more complex eating behaviors, including other CNS sites that are sufficient for and/or required for ghrelin’s orexigenic actions. Notwithstanding these as-of-yet unanswered questions, the data here does add significantly to our understanding of the actions of ghrelin, highlighting the distributed nature of ghrelin signaling within the CNS.
